# Evaluating Wharton's Jelly-Derived Mesenchymal Stem Cell's Survival, Migration, and Expression of Wound Repair Markers under Conditions of Ischemia-Like Stress

**DOI:** 10.1155/2017/5259849

**Published:** 2017-02-07

**Authors:** Iris Himal, Umesh Goyal, Malancha Ta

**Affiliations:** Indian Institute of Science Education and Research, Kolkata, West Bengal, India

## Abstract

The efficacy of mesenchymal stem cell (MSC) therapy is currently limited by low retention and poor survival of transplanted cells as demonstrated by clinical studies. This is mainly due to the harsh microenvironment created by oxygen and nutrient deprivation and inflammation at the injured sites. The choice of MSC source could be critical in determining fate and cellular function of MSCs under stress. Our objective here was to investigate the influence of ischemia-like stress on Wharton's jelly MSCs (WJ-MSCs) from human umbilical cord to assess their therapeutic relevance in ischemic diseases. We simulated conditions of ischemia in vitro by culturing WJ-MSCs in 2% oxygen in serum deprived and low glucose medium. Under these conditions, WJ-MSCs retained viable population of greater than 80%. They expressed the characteristic MSC surface antigens at levels comparable to the control WJ-MSCs and were negative for the expression of costimulatory molecules. An upregulation of many ECM and adhesion molecules and growth and angiogenic factors contributing to wound healing and regeneration was noted in the ischemic WJ-MSC population by a PCR array. Their migration ability, however, got impaired. Our findings provide evidence that WJ-MSCs might be therapeutically beneficial and potent in healing wounds under ischemic conditions.

## 1. Introduction

Mesenchymal stem cells, located in the perivascular niches, are multipotent cells which have a broad tissue distribution and in the last few years have already been isolated from a wide variety of tissues such as bone marrow, skin, deciduous tooth, menstrual blood, adipose tissue, and umbilical cord [1]. Though MSCs from the different tissue sources share some basic biological characteristics, many of them have been reported to differ from each other with respect to their phenotypic, differentiation, transcriptomic, and proteomic profiles, thus being assigned their distinct molecular signatures [2]. It is the origin or source of MSCs that determines their fate and molecular and functional characteristics. The umbilical cord, an abundantly available and discarded tissue source of MSCs, is a suitable and convenient alternative to the bone marrow (BM) as its collection does not involve any invasive procedures or major ethical concerns [3]. Wharton's jelly is the collagenous, connective tissue surrounding the umbilical cord vessels [4] and being of fetal origin, WJ-derived MSCs are considered to be more primitive and possess characteristics between embryonic and adult stem cells [5].

The regenerative and therapeutic effects of MSCs are majorly due to their immunomodulatory and/or trophic roles. MSCs are thought to create a protective curtain of immunomodulation, while a parallel process of tissue regeneration takes place in the region of injury or inflammation, both mediated via the secretion of bioactive molecules [6]. In addition, MSCs have self-renewal and differentiation capacity [7], migration and homing properties [8], and immunoprivileged status [9], all of which together make them an attractive option for cell-based therapeutic applications.

However, in spite of the glowing promises, MSC-based treatments have been unfortunately found to be marginally successful and the success is mainly hindered by low retention and survival rate of transplanted MSCs and their poor engraftment in the injured tissues [10–12]. To deliver optimal clinical benefits, transplanted MSCs need to survive, migrate, and home efficiently in sufficient numbers and be functionally active in pathological areas in vivo. The pathological areas or damaged tissues under conditions such as stroke, myocardial infarction, and spinal and critical limb ischemia have an inhospitable microenvironment marked by prevalence of low oxygen tension and nutrient deprivation along with other complexities. A majority of the grafted MSCs have been reported to die soon after transplantation into the ischemic region. Two possible factors which could be responsible for this are as follows. First, the clinical grade MSCs used for transplantations are usually expanded in vitro in controlled environment in the presence of serum rich medium and atmospheric oxygen tension which is very different from the harsh ischemic pathological conditions in vivo. Second, most of these studies have been carried out with BM-MSCs. MSCs from other tissue sources might behave differently and exhibit differences in therapeutic potential under ischemic tissue environment, thus, leading to an altogether different outcome. Hence, taking the above into consideration, the present study was designed to investigate the impact of ischemia-like stress conditions created in vitro, first, on certain primary characteristics such as morphology, viability, and immunophenotype and next, on some clinically relevant properties such as immune-privilege status, migration ability, and expression levels of wound healing and regeneration markers of umbilical cord-derived WJ-MSCs [13].

## 2. Materials and Methods

### 2.1. WJ-MSC Isolation and Culture

Fresh human umbilical cords (*n* = 6) were collected after full-term births (cesarean section or normal vaginal delivery) with informed consent using the guidelines approved by the Institutional Ethics Committee and Institutional Committee for Stem Cell Research and Therapy (IC-SCRT) at the Indian Institute of Science Education and Research (IISER), Kolkata, India. WJ-MSCs from umbilical cords were isolated by the explant culture method as previously described [14]. Briefly, umbilical cord segments were slit longitudinally, cord vessels were manually removed, and the underlying perivascular tissue was cut into small explants and directly placed in 35 mm tissue culture plastic dishes (Falcon, BD Biosciences, San Jose, CA) in KnockOut Dulbecco's Modified Eagle's Medium with 4.5 g/L glucose (KnockOut DMEM; Life Technologies, Grand Island, NY), 2 mM L-glutamine (Life Technologies), supplemented with 10% fetal bovine serum (FBS, MSC-qualified; Life Technologies), and 1x Antibiotic-Antimycotic (Life Technologies). After 7–10 days, once cells started to appear, explants were removed. On reaching 70–80% confluency, cells were dissociated and replated. Following passage 0 (P0), in all the subsequent passages WJ-MSCs were cultured in the same above-mentioned medium except that anti-anti was replaced with 1% penicillin streptomycin (Life Technologies). All cell dissociations were carried out with TrypLE Express (Life Technologies) and cells were plated at 5000 cells/cm^2^. All the experiments were performed with WJ-MSCs between passages 4 and 6. For standard control conditions, WJ-MSC cultures were incubated at 37°C with 5% humidified CO_2_ and 20% O_2_.

To mimic conditions of ischemia, WJ-MSCs were first plated and allowed to grow in control conditions for 48–72 hours and next were transferred to a tri-gas incubator flushed with humidified gas mixture of composition 2% O_2_- 5% CO_2_- 93% N_2_ and the culture medium was replaced with L-glutamine containing DMEM-low glucose medium (1 g/L glucose) (Life Technologies,) supplemented with 1% penicillin streptomycin. In the ischemic conditions, cultures were maintained for 48 hours. In case of long-term ischemia, cultures were maintained under the ischemic conditions for 5-6 days.

### 2.2. Growth Kinetics

For the calculation of population doubling time, WJ-MSCs were seeded at a density of 5000 cells/cm^2^. Control WJ-MSCs were harvested after 72 hrs, while ischemic WJ-MSCs were exposed to ischemia-like stress conditions for 48 hrs after an initial incubation under control conditions for 24 hrs. The population doubling time was obtained by the formula: TD = *t∗*log_10_⁡2/(log_10_⁡NH − log_10_⁡NI). NI: the inoculum cell number; NH is the cell harvest number, and *t* is the time of the culture (in hours) [15].

### 2.3. Apoptosis Assay

To test and compare for the presence of apoptotic cells between control and ischemic WJ-MSCs, we used the Alexa Fluor® 488 Annexin-V/Dead Cell Apoptosis Kit (Life Technologies, Eugene, Oregon, USA) as per the manufacturer's instructions. Externalization of phosphatidylserine on the outer leaflet of the plasma membrane of the apoptotic cells was detected by annexin-V while PI labelled necrotic and late apoptotic cells with permeabilized membranes. After staining the cells with Annexin-V Alexa Flour 488 and PI, the samples were analyzed by BD FACSCalibur flow cytometry (BD Biosciences, San Jose, CA, USA). Compensation controls were included in every experiment.

### 2.4. MTT Assay

WJ-MSCs were plated in a 96-well plate and grown under standard control conditions for 24 hrs. Next, ischemia-like condition was initiated and after 48 hrs of incubation under respective control and ischemia conditions, MTT (Sigma-Aldrich) was added at a final concentration of 1 mg/mL and incubated at 37°C for 3 hours. Finally reaction was terminated by removing MTT and adding DMSO to solubilize the formazan formed. Absorbance was recorded at 595 nm in an ELIZA plate reader. Proliferation was expressed as ratio of OD of ischemic sample to OD of control sample.

### 2.5. Senescence Assay

Senescence assay was performed with WJ-MSCs cultured on 35 mm dishes using Senescence Cells Histochemical Staining Kit (Sigma-Aldrich), according to the manufacturer's instructions. Briefly, cells were washed with phosphate-buffered saline, fixed and stained using staining solution containing X-gal. The dishes were next incubated overnight at 37°C without CO_2_. Cells were observed for development of blue colour under Olympus BX-51 microscope (Olympus, Tokyo, Japan) and images were captured using the DP25 digital camera (Olympus). Three random fields each from three different WJ-MSC long-term ischemia and control cultures were used to calculate the percentage of blue cells.

### 2.6. Immunophenotypic Characterization

Control and ischemic WJ-MSCs were characterized for surface antigen expression by flow cytometry analysis. Anti-human antibodies such as CD90-PE, CD73-PE, CD105-PE, CD34-PE, CD80-PE, and CD40-PECy7 were used to mark the cell surface markers (all from BD Pharmingen, SanDiego, CA, USA). Mouse isotype antibodies served as control (BD Pharmingen). At least 10,000 events were acquired on BD FACSCalibur flow cytometer and analyzed with BD CellQuest Pro software.

### 2.7. In Vitro Scratch Assay

To evaluate migration in vitro under conditions of ischemia-like stress, WJ-MSCs were cultured under standard control and ischemic conditions and on reaching complete confluency, culture monolayers were scraped with the help of a pipette tip to create scratch of uniform width. Migration of WJ-MSCs into the scratch area was assessed by photographic images collected at different time points after scratch till wound closure using the 10x objective of Olympus Phase Contrast Microscope (Olympus IX-81; Olympus, Tokyo, Japan). Migration rate was calculated by measuring the distance between the two edges of the scratch at 2 h and at 9 hr and dividing by the time interval. Measurements were carried out at ten different points for each scratch.

### 2.8. cDNA Synthesis and Human Wound Healing PCR Array

Total RNA was isolated from three samples each of ischemic and their corresponding control WJ-MSCs (*n* = 3) using RNeasy Plus Mini Kit (Qiagen, Hilden, Germany) according to the manufacturer's instructions. RNA yield was quantified using Nanodrop ND-1000 (Thermo Scientific, Waltham, MA, USA) and equal amounts of RNA samples were pooled from the three different sets for ischemic and the corresponding control WJ-MSCs. Complementary DNA (cDNA) of the pooled RNA sample was synthesised using RT^2^ First Strand Kit (Qiagen Sciences, Maryland, USA) according to manufacturer's instructions. cDNA was mixed with SYBR Green/ROX qPCR Master Mix (Qiagen Sciences), and 25 *μ*L aliquots were loaded into each well of the Human Wound Healing RT^2^ Profiler™ PCR Array System (Qiagen Sciences). Array run was performed on an ABI Biosystems StepOnePlus (Applied Biosystems, Carlsbad, CA), and StepOnePlus version v.2.2 software (Applied Biosystems) was used to analyze the results. Samples with a cycle threshold of ≤35 were taken for calculating the fold change in expression. The arithmetic mean of five housekeeping genes was used to normalize the data (Ct = Ct gene − mean Ct housekeeping).

### 2.9. RT-PCR Confirmation of Array Data

To validate the gene expression profile obtained from the Human Wound Healing RT^2^ Profiler PCR Array, a few randomly selected genes from the array were subjected to semiquantitative RT-PCR analysis. cDNA was prepared from three donor pooled RNA samples of ischemic and control WJ-MSCs, respectively, using RT^2^ First Strand Kit according to manufacturer's instructions. PCR amplification was performed using Taq DNA polymerase (Invitrogen, Carlsbad, CA, USA) to compare the expression. GAPDH was used as an internal control. The primer sequences used in the reverse transcriptase PCR analysis and product size are listed in Table 1(a).

### 2.10. Statistical Analysis

Data are presented as mean ± standard error of the mean. Graphical representations and data analysis were performed using GraphPad Prism 5 software (GraphPad, USA). Statistical comparisons were made using two-tailed Student's *t*-test. *p* values ≤ 0.05 were considered significant.

## 3. Results

### 3.1. Morphological Characteristics and Proliferation under Ischemia-Like Stress

**Figure 1:**
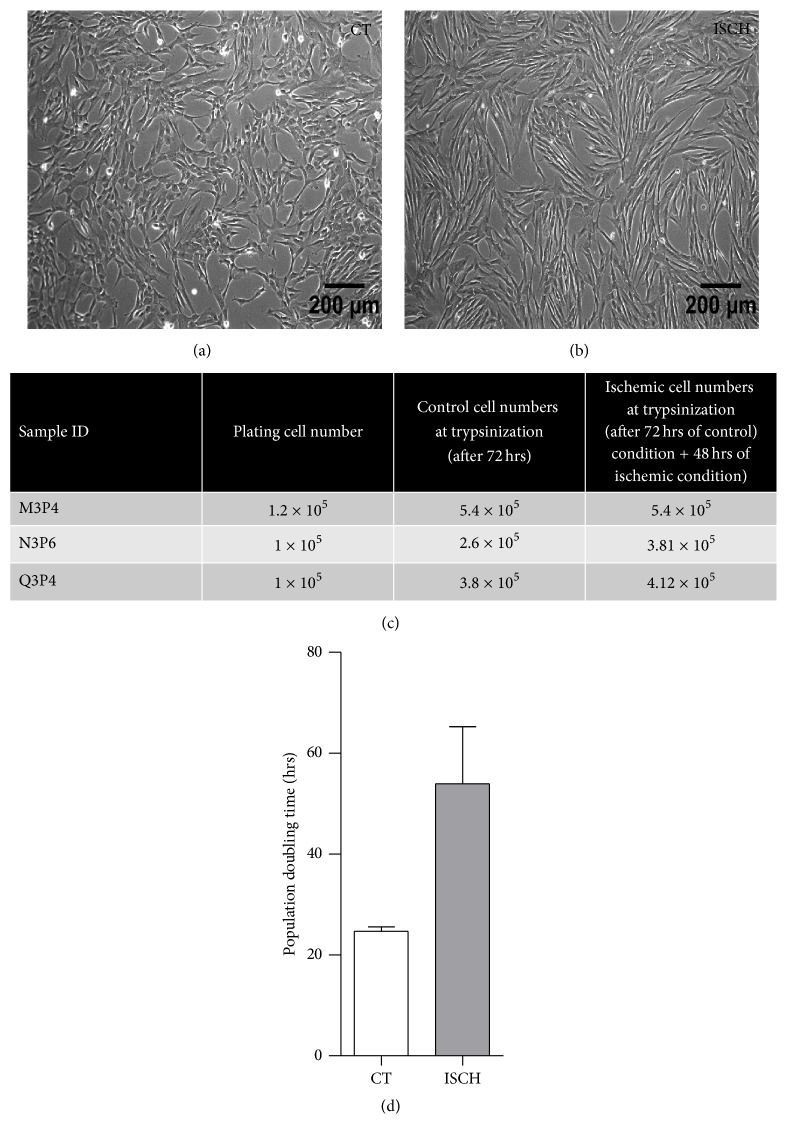
Effect of simulated ischemic stress on morphology of WJ-MSCs. Morphology of WJ-MSCs maintained under control conditions (a) and ischemic stress conditions mimicked by exposing WJ-MSCs to hypoxia (2% O_2_), low glucose, and serum-free medium for 48 hours (b). Representative phase contrast images from three independent biological samples of WJ-MSC (*n* = 3) are presented. Comparison of total cell yield between control and ischemic WJ-MSCs. All cells were plated at a density of 5000 cells/cm^2^ and grown under control conditions for 72 hours. After 72 hours, control cultures were harvested while ischemic cultures were shifted to ischemia conditions for another 48 hours and then harvested (*n* = 3) (c). Comparison of population doubling time between control and ischemic WJ-MSCs (d). Results represent the average of three independent cultures with SEM (Student's *t*-test, two tailed).

To determine the effect of ischemia-like stress on some basic characteristics of WJ-MSCs, conditions of ischemia were simulated in vitro by exposing cells to a combination of 2% oxygen, low glucose, and no serum for 48 hrs. Ischemic WJ-MSCs adopted a thin, elongated morphology as compared to the control cells which maintained the typical spindle-shaped fibroblast-like morphology (Figures 1(a) and 1(b)). Estimation and comparison of cell numbers harvested at the end of control conditions versus cell numbers obtained after an additional 48 hours under conditions of ischemia-like stress are shown in Figure 1(c). Population doubling times of 24.6 ± 0.9 and 53.9 ± 11.3 hr for control and ischemia-like conditions, respectively (*p* = 0.06), indicated that the ischemic cells had reduced proliferation rate (Figure 1(d)). MTT assay indicated that ischemic WJ-MSCs had approximately 70% proliferation rate in comparison to the control WJ-MSCs (data not shown).

### 3.2. Evaluation of Viability under Conditions of Ischemia-Like Stress

**Figure 2:**
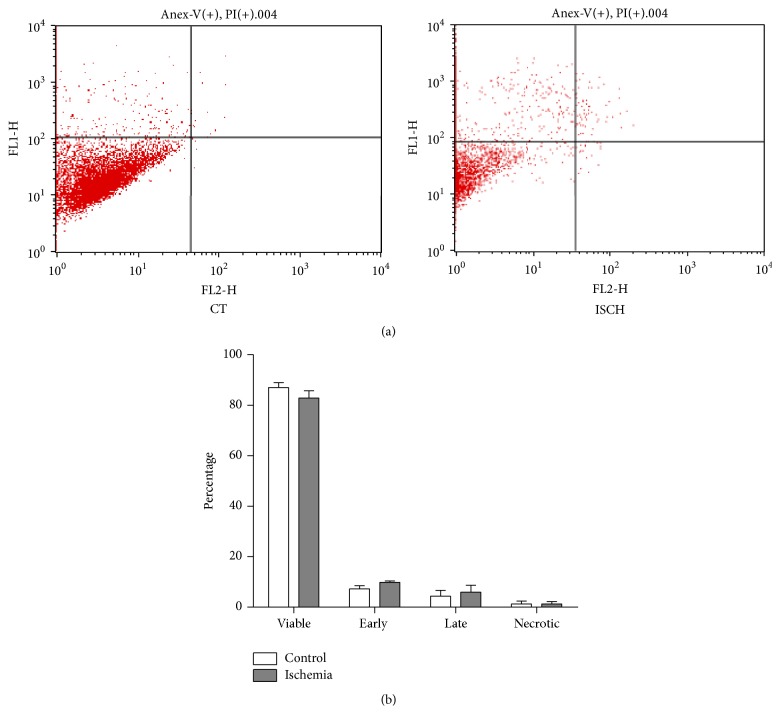
Detection of apoptosis by flow cytometry. Quantitation of live, early apoptotic, late apoptotic, and necrotic population of WJ-MSCs under control and ischemia-like stress conditions (a). Comparison of the percentages of live, apoptotic (early and late), and necrotic populations between control and ischemic WJ-MSC cultures as depicted by the histogram. Each bar represents mean ± standard error of the mean (*n* = 3) (b). Data shown are representative of at least three independent biological samples.

Next, to assess if simulated conditions of ischemia induced cell death in WJ-MSCs, cell apoptosis was measured by annexin-V-FITC and the percentages of annexin-V+/PI− and annexin-V+/PI+ cells were determined and compared between control and ischemic WJ-MSCs using flow cytometry (Figure 2(a)). Annexin-V+/PI− population marked the early apoptotic cells while annexin-V+/PI+ labelled the late apoptotic/necrotic population. After 48 hrs of exposure to ischemia, ischemic WJ-MSCs exhibited 9.8 ± 0.5/5.9 ± 2.7 early/late apoptotic cells as against 7.2 ± 1.2/4.3 ± 2.2 for control WJ-MSCs. The percentages of viable cells were 82.8 ± 2.8 and 87 ± 1.9 for ischemic and control WJ-MSCs, respectively (Figure 2(b)). The differences were not significant.

### 3.3. Surface Phenotype Comparison between Control and Ischemic WJ-MSCs

**Figure 3:**
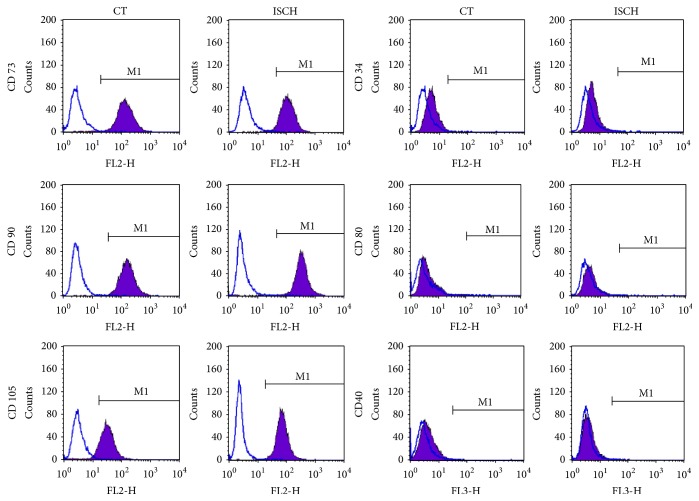
Comparison of CD surface marker profile between control and ischemic WJ-MSCs by flow cytometry. WJ-MSCs, cultured under control and ischemic conditions, were labelled with the indicated antibodies and analyzed by flow cytometry. Open area represents antibody isotype control for background fluorescence while the shaded area represents positive reactivity with the indicated antibodies. Representative histograms are depicted. Results are representative of at least 3-4 independent biological samples.

To compare the immunophenotype profile of WJ-MSCs cultured under ischemia-like stress versus standard culture conditions, the two populations were assessed for the expression of MSC surface antigens. Flow cytometry analysis revealed that both the populations of MSCs were positive for CD 73, CD 90, and CD 105 and expressed these markers equally, while they were negative for CD 34 (Figure 3).

Under normal circumstances, human MSCs express low level of MHC class I and lack the expression of MHC class II and the classical costimulatory molecules, which explains their immune-privileged status [9]. Just to confirm, we tested for the expression of costimulatory molecules CD80 and CD 40 under in vitro simulated ischemic stress conditions. No difference was detected between the two populations and both control and ischemic WJ-MSCs were found to be negative for their expression, as demonstrated by flow cytometry (Figure 3).

### 3.4. Influence of Ischemia on Migration of WJ-MSCs

**Figure 4:**
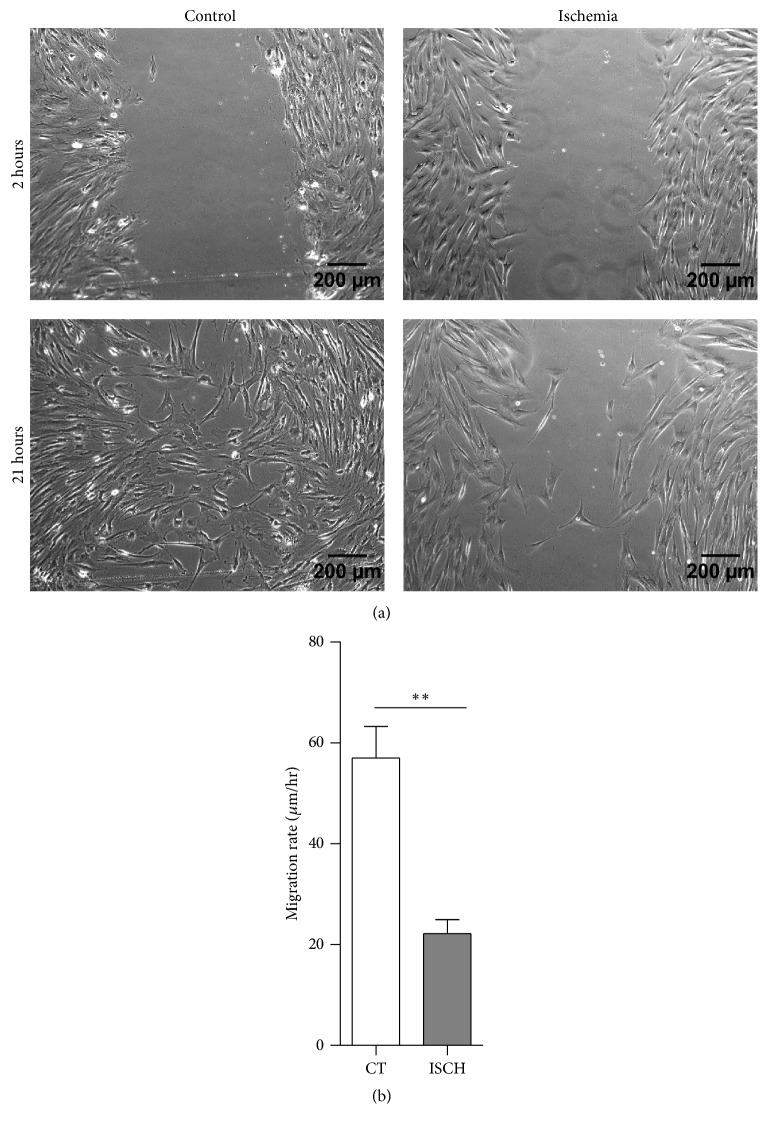
Comparison of scratch induced migration between control and ischemic WJ-MSCs. Representative images of migration at 2 and 21 hours after scratch are shown for control and ischemic WJ-MSCs (a). Average migration rate during the first 9 hrs in response to the scratch was calculated from three independent experiments (*n* = 3). Bars represent mean ± SEM (Student's *t*-test, two tailed, *∗∗* represents *p* < 0.01) (b).

To explore the influence of ischemia-like stress on migration of WJ-MSCs, an in vitro scratch wound healing assay was performed where a scratch of uniform width was made in area of confluent monolayer in control and ischemic dishes of WJ-MSCs and their migration during scratch closure was studied and compared over a period of 21 hours (Figure 4(a)). Ischemic WJ-MSCs were slower to migrate and took longer to heal the scratch as compared to control WJ-MSC cells. The migration rate, calculated from the scratch assay, was found to be 56.9 ± 6.2 *μ*m/hr for control WJ-MSCs while that for ischemic WJ-MSCs was 22.1 ± 2.8 *μ*m/hr (*p* = 0.007) (Figure 4(b)).

### 3.5. Effect of Long-Term Ischemia-Like Stress on WJ-MSCs

**Figure 5:**
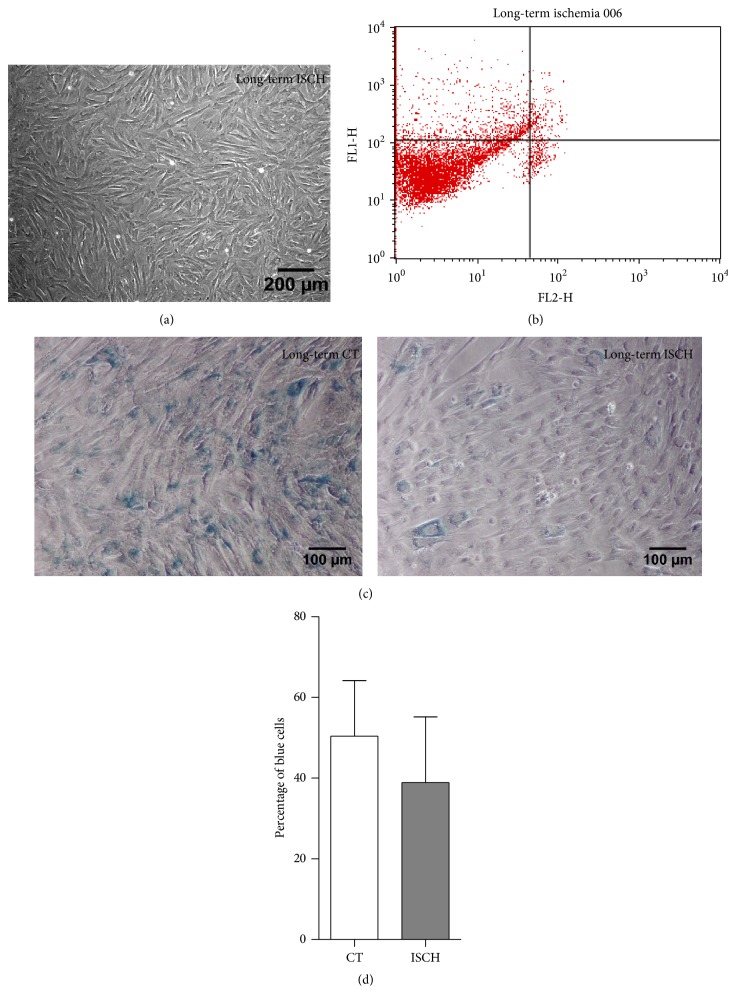
Effect of long-term ischemia-like stress on morphology and viability of WJ-MSCs. The time course of exposure to ischemic conditions was extended to 5-6 days. Morphology of WJ-MSCs after being maintained under long-term ischemia (a). Flow cytometry analysis of apoptotic WJ-MSCs stained with annexin-V-PI following exposure to long-term ischemia (b). Senescence, as detected by senescence-associated *β*-galactosidase activity, in both long-term ischemia and control cultures (c). Quantification of the percentage of SA-*β*-gal positive blue cells of long-term ischemia and control WJ-MSC (d). Results are representative of at least three independent biological samples.

Next, WJ-MSCs were exposed to the simulated ischemic stress conditions for a continuous period of 5-6 days. The cell morphology did not show much difference (Figure 5(a)). A longer term exposure to ischemia-like stress condition still resulted in 79.9 ± 2.5% of viable WJ-MSCs, while the early/late apoptotic cells were detectable at 17.5 ± 1.7/1.7 ± 0.6% by apoptosis assay (Figure 5(b)). Though there was a distinct increase in senescent population from the 48 hrs time point, it is noteworthy that strong senescence induction was also observed in control WJ-MSC cultures which were kept in culture without subculturing for 5-6 days (Figure 5(c)). The difference in the percentage of senescence-associated *β*-galactosidase (SA-*β*-gal) positive cells was not found to be significant between long-term control and ischemic WJ-MSCs (Figure 5(d)).

### 3.6. Ischemia-Like Stress and Expression of Wound Healing Related Genes as Demonstrated by PCR Array

**Figure 6:**
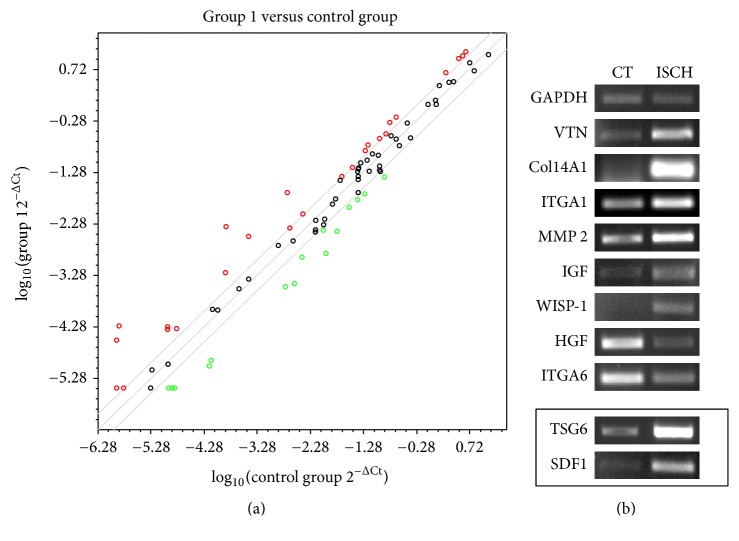
Changes in wound healing related gene expression as evidenced by PCR array. Scatterplot comparing wound healing related gene expression profile between control and ischemic WJ-MSCs. Genes with at least 2-fold differential expression are depicted (a). RT-PCR validation of a few randomly selected genes from the wound healing RT^2^ Profiler PCR Array. PCR was performed using complementary cDNA pooled from three different samples, each of ischemic and their corresponding control WJ-MSC cultures. GAPDH was used as an internal control. TSG6 and SDF1, which were not part of the array, are shown in the box (b).

MSCs are known to create regenerative microenvironment in an injured tissue by secreting bioactive factors which not only have immunoregulatory role but also aid in wound healing and repair. Wound healing requires a tight coordination between cells, ECM protein and growth factors. Hence, to evaluate the effect of ischemia-like stress on the wound healing properties of WJ-MSCs, we compared the gene expression profile of a set of wound healing related genes between control and ischemic WJ-MSCs. The human wound healing RT^2^ profiler PCR array system was used to analyze and compare the expression profiles. RNA from three different samples of WJ-MSCs treated under conditions of ischemia for 48 hrs and their corresponding controls were pooled, and a single replica was run for each condition. This array profiles the expression of 84 critical genes which are important in the wound healing response (Table 1(b)). We focused on genes with greater than or equal to twofold change in expression to evaluate transcriptional changes between control and ischemic WJ-MSCs and a scatterplot of the data is displayed in Figure 6(a). Many of the genes belonging to collagen family were found to be upregulated, such as COL141, COL1A1, COL1A2, COL3A1, COL41, COL4A3, and COL5A1 (Table 2). MSCs act as trophic mediators and some of the trophic factors found to be upregulated in ischemic WJ-MSCs were IGF1, VEGFA, and PDGFA (Table 2). There was also an upregulation of some key anti-inflammatory/immunomodulatory cytokine genes such as IL4, IL6, SDF1, and TSG6. The downregulated genes comprised of some inflammatory molecules such as TNF*α* and IL-2 and certain chemokines like CXCL1, CXCL2, CCL2, and CCl7. Also, HGF was found to be strongly downregulated under conditions of ischemic stress in WJ-MSCs (Table 2).

### 3.7. Reverse Transcriptase-Polymerase Chain Reaction (RT- PCR) Confirmation of PCR Array Data

Semiquantitative RT- PCRs, using pooled cDNA samples used in the array (Figure 6(b)) and cDNA from individual samples that constituted the pool (data not shown), confirmed the fidelity of the array data. The differential expression patterns observed in the array between control and ischemic WJ-MSCs were in good agreement with RT-PCR data for the few randomly selected genes tested (Figure 6(b)).

The gene expression comparison for TSG6 and SDF1, which were not included in the PCR array, was obtained by semiquantitative RT-PCR (Figure 6(b), boxed panel).

## 4. Discussion

MSC-based cell therapy has been hampered by poor survival rates of transplanted MSCs, which is mainly attributed to the prevalence of hostile microenvironment, created by ischemia and inflammation, at the injury sites. Prolonging the persistence of viable and functionally active MSCs would directly lead to enhanced efficacy of MSC-based treatments and this could depend on the source of MSCs [16]. Thus, our study here is targeted at highlighting the potential advantage and usefulness, if any, of WJ-MSCs from the umbilical cord in the treatment of ischemic diseases.

The in vivo ischemic state is complex and difficult to replicate; however, an attempt was made to simulate certain aspects in vitro by subjecting WJ-MSCs to 2% hypoxia in a serum deprived and low glucose medium. Though the proliferation of ischemic WJ-MSCs reduced as confirmed from cell numbers and MTT assay, they survived 48 hrs of exposure to ischemia-like stress condition with greater than 80% viable cells, which was comparable to the viable cell numbers under standard control culture conditions.

Besides viability, the ischemic WJ-MSCs continued to express all the MSC-characteristic surface antigens at comparable levels to control WJ-MSCs, suggesting that oxygen, serum, or glucose levels do not influence the immunophenotype profile of WJ-MSCs. This was confirmed independently with just serum deprived WJ-MSCs as well (data not shown). These data are in contrast to a previous report [17] where the authors observed a significant reduction in immunophenotypic marker expression and viability of WJ-MSCs under their conditions of in vitro ischemia. This is not very unexpected considering the differences in the protocols adopted for isolation and expansion of tissue-derived MSCs between different laboratories across the world and hence the enormous variations in heterogeneity and quality between the cells [18]. Our ischemic WJ-MSCs were also found negative for the expression of costimulatory molecules, confirming their low immunogenicity status even under stress conditions. Overall, our viability and immunophenotype data for ischemic WJ-MSCs are quite encouraging. Moreover, even after a long-term episode of continuous ischemic stress for 5-6 days, viability was maintained at >75%.

Efficient migration to the target site of injury, following transplantation, is another critical parameter for a successful regenerative response. Hence, we were keen to evaluate the movement of WJ-MSCs in response to ischemia-like stress. Surprisingly, ischemic WJ-MSCs exhibited decreased migrational ability as compared to control cells. To further explore the specific stress condition which led to the decreased migration, WJ-MSCs were cultured under the individual stress conditions and an in vitro scratch assay was performed. Serum deprivation was identified to be responsible for the reduced mobility of the MSCs with migration rate for control and no serum WJ-MSCs being 59 ± 5 and 34.2 ± 5.4 *μ*m/hr, respectively (*p* = 0.02) (Supplemental Figure S1A in Supplementary Material available online at https://doi.org/10.1155/2017/5259849). Only hypoxia condition led to increased mobility, though not significant (*p* = 0.08) and only low glucose medium did not affect migration of WJ-MSCs (Supplemental Figures S1A, B). To our knowledge this is the first report of effect of ischemic stress on stem cell migration and needs to be investigated further to improve the mobility of MSCs in ischemic environment.

Wound healing is a complex, multistep process involving active coordination between cells, growth factors, and ECM proteins [19]. We were curious to investigate the wound healing capacity of WJ-MSCs under ischemic stress conditions at the transcription level. Our PCR array data revealed an upregulation of many of the collagen family genes such as COL141, COL1A1, COL1A2, COL3A1, COL41, COL4A3, and COL5A1 in the ischemic WJ-MSCs as compared to control cells. Similarly, there was an increase in the expression of VTN which again is an adhesive protein found in the extra cellular matrix. This increase in expression in the matrix genes is promising as remodelling of ECM along with collagen deposition is a crucial aspect of normal wound healing [19]. This could also be the reason why we repeatedly found ischemic WJ-MSCs taking longer to detach than control cells during trypsinization (data not shown). During the wound healing process, usually there is a decrease in the secretion of metalloproteinases responsible for matrix breakdown with a simultaneous increase in the production of tissue inhibitor of metalloproteinases. We observed a twofold increase in TIMP1 and a threefold downregulation in MMP1 in ischemic WJ-MSCs, although there was an upregulation of MMP2 in the ischemic WJ-MSCs. WISP1, a matricellular protein which regulates various pathways to alter processes such as cell migration, angiogenesis, mitosis, apoptosis, and ECM production, was also found to be strongly upregulated in ischemic WJ-MSCs [20].

The trophic role of MSCs involves release of an array of growth factors and cytokines which induce cell proliferation and angiogenesis [21]. Interestingly, under conditions of ischemia we noted a higher expression of IGF1, VEGFA, and PDGFA as compared to control WJ-MSCs. While IGF1 and VEGF both play a role in recruiting endothelial cells and stimulating local angiogenesis, IGF1 is also known to function as a mitogen and increase fibroblast, epithelial, and endothelial cell division [21]. The paracrine mechanisms of MSCs also include secretion of bioactive molecules with anti-inflammatory and immunomodulatory roles such as, TGF-b1, HGF, SDF1, TSG6, prostaglandin 2, NO, IDO, IL4, IL6, and IL10 [21]. From our PCR array data, while we found a 2-fold or greater increase in the gene expression levels of IL4 and IL6 in ischemic WJ-MSCs as compared to control MSCs, there was a reduced expression of some of the inflammatory molecules such as TNF-a and IL2 in ischemic WJ-MSCs. HGF which usually mediates antifibrotic effects and acts as a mobilizing factor on the contrary showed a downregulation in WJ-MSCs under ischemia [22].

Though not part of our wound healing PCR array, we found a strong upregulation of two important genes, TSG6 and SDF1, in ischemic WJ-MSCs by semiquantitative RT-PCR. TSG6 is an inflammation-modulating protein which has multiple anti-inflammatory actions [23] and in several animal models of injury, TSG6, by itself, has been found to be sufficient to compensate for the therapeutic activity of MSCs [24]. SDF1 plays a critical role in wound healing by its antiscarring property as well as its ability to recruit and stimulate proliferation of tissue resident stem or progenitor cells [16, 21]. Our conditions of ischemia led to downregulation of certain chemoattractant molecules, such as CXCL1, CXCL2, CCL2, and CCL7, which primarily increase migration of MSCs and cells of the monocyte-macrophage system [25, 26]. This could be in support of our migration data for ischemic WJ-MSCs. CCL2 and CCL7 also have an inhibiting effect on immunoglobulin production by plasma cells [26].

Taken together, our data reflects that WJ-MSCs are well equipped to handle ischemia-like stress and many of the MSC-characteristic properties of WJ-MSCs remain unaffected under ischemia. In fact, in support of our findings, a previous report had even demonstrated genetic stability of umbilical cord MSCs under various hostile in vivo-like conditions, including ischemia [27].

## 5. Conclusions

This is the first report, to our knowledge, demonstrating the influence of ischemia-like stress on migration and expression profile of wound healing related genes in WJ-MSCs. As a future direction, the trophic and immunomodulatory factors secreted by WJ-MSCs and the mechanisms involved in migration impairment under conditions of ischemia will be analyzed. WJ-MSCs seem to have the ability to withstand harsh conditions of ischemia-like stress and need to be considered more seriously as a promising source for clinical applications.

## Supplementary Material

Effect of individual stress conditions on migration of WJ-MSCs. As ischemic WJ-MSCs exhibited reduced migrational ability compared to control WJ-MSCs, to further investigate the specific stress condition responsible for the decreased migration, WJ-MSCs were cultured under the individual stress conditions and an in vitro scratch assay was performed.

## Figures and Tables

**Table 1a:** (a) Primer sequences used for semiquantitative RT-PCR analysis

S. number	Gene name	Forward primer	Reverse primer	Size (bp)
1	IGF-1	5′ TCTTGAAGGTGAAGATGCACACCA 3′	5′ AGCGAGCTGACTTGGCAGGCTTGA 3′	303
2	COL14A1	5′ AAGCCCAGAGTCAAAGTTGTGGA 3′	5′ CCATGAACCATCGACCAGGA 3′	123
3	MMP 2	5′ GGCCCTGTCACTCCTGAGAT 3′	5′ GGCATCCAGGTTATCGGGGA 3′	473
4	ITGA1	5′ GGTTCCTACTTTGGCAGTATT 3′	5′ AACCTTGTCTGATTGAGAGCA 3′	149
5	ITGA6	5′ TCCCTGAACCTAACGGAGTCT 3′	5′ ATGTCCAAGTAGTTCAGTTTG 3′	253
6	SDF-1	5′ ATGAACGCCAAGGTCGTGGTC 3′	5′ CTTGTTTAAAGCTTTCTCCAGGTACT 3′	267
7	WISP-1	5′ AGAGCCGCCTCTGCAACTT 3′	5′ GGAGAAGCCAAGCCCATCA 3′	245
8	GAPDH	5′ GAGTCAACGGATTTGGTCGT 3′	5′ TTGATTTTGGAGGGATCTCG 3′	248
9	VTN	5′ CGAGGAGAAAAACAATGCCAC 3′	5′ GAAGCCGTCAGAGATATTTCG 3′	498
10	TSG 6	5′ CCCATTGTGAAGCCAGGGCCCAACTG 3′	5′ GGAAGCTCATCTCCACAGTATCTTCCC 3′	361
11	HGF	5′ TCACGAGCATGACATGACTCC 3′	5′ AGCTTACTTGCATCTGGTTCC 3′	302

**Table 1b:** (b) Human wound healing-related genes screened using the RT^2^ Profiler PCR Array

ACTA2	ACTC1	ANGPT1	CCL2	CCL7	CD40LG	CDH1	COL14A1	COL1A1	COL1A2	COL3A1	COL4A1
COL4A3	COL5A1	COL5A2	COL5A3	CSF2	CSF3	CTGF	CTNNB1	CTSG	CTSK	CTSV	CXCL1
CXCL11	CXCL2	CXCL5	FGF	EGFR	F13A1	F3	FGA	FGF10	FGF2	FGF7	HBEGF
HGF	IFN-G	IGF1	IL10	IL1B	IL2	IL4	IL6	IL6ST	ITGA1	ITGA2	ITGA3
ITGA-4	ITGA-5	ITGA-6	ITGAV	ITGB1	ITGB3	ITGB5	ITGB6	MAPK1	MAPK3	MIF	MMP1
MMP2	MMP7	MMP9	PDGFA	PLAT	PLAU	PLAUR	PLG	PTEN	PTGS2	RAC1	RHOA
SERPINE1	STAT3	TAGLN	TGFA	TGFB1	TGFBR3	TIMP1	TNF	VEGFA	VTN	WISP1	WNT5A
ACTB^a^	B2M^a^	GAPDH^a^	HPRT1^a^	RPLP0^a^	HGDC^b^	RTC^c^	RTC^c^	RTC^c^	PPC^d^	PPC^d^	PPC^d^

^a^House keeping genes. ^b^Human genomic DNA contamination control.

^c^Reverse transcription control. ^d^Positive PCR control.

**Table 2a:** (a) Genes ≥ 2.0-fold in ischemic WJ-MSCs as compared to control WJ-MSCs

Number	Gene symbol	Gene description	Fold difference
1.	ACTA2	Actin, alpha 2, smooth muscle, aorta	2.4
2.	COL14A1	Collagen, type XIV, alpha 1	33.1
3.	COL1A1	Collagen, type I, alpha 1	2.59
4.	COL1A2	Collagen, type I, alpha 2	2.5
5.	COL3A1	Collagen, type III, alpha 1	2.6
6.	COL4A1	Collagen, type IV, alpha 1	2.0
7.	COL4A3	Collagen, type IV, alpha 3 (Goodpasture antigen)	4.3
8.	COL5A1	Collagen, type V, alpha 1	2.99
9.	CTSK	Cathepsin K	2.1
10.	CXCL11	Chemokine (C-X-C motif) ligand 11	24.6
11.	IGF1	Insulin-like growth factor 1 (somatomedin C)	41.4
12.	IL4	Interleukin 4	3.05
13.	IL6	Interleukin 6 (interferon, beta 2)	1.9
14.	ITGA1	Integrin, alpha 1	2.4
15.	MMP2	Matrix metallopeptidase 2 (gelatinase A, 72 kDa gelatinase, 72 kDa type IV collagenase)	2.84
16.	PDGFA	Platelet-derived growth factor alpha polypeptide	2.02
17.	PTEN	Phosphatase and tensin homolog	2.13
18.	TGFA	Transforming growth factor, alpha	4.9
19.	TIMP1	TIMP metallopeptidase inhibitor 3	2.1
20.	VGEFA	Vascular endothelial growth factor A	2.3
21.	VTN	Vitronectin	11.05
22.	WISP1	WNT1 inducible signaling pathway protein 1	8.48

**Table 2b:** (b) Genes downregulated ≥ 2.0 in ischemic WJ-MSCs as compared to control WJ-MSCs

Number	Gene symbol	Gene description	Fold difference
1.	CCL2	Chemokine (C-C motif) ligand 2	−2.33
2.	CCL7	Chemokine (C-C motif) ligand 7	−7.21
3.	CSF2	Colony stimulating factor 2 (granulocyte-macrophage)	−3.32
4.	CTSV	Cathepsin L2	−5.52
5.	CXCL1	Chemokine (C-X-C motif) ligand 1 (melanoma growth stimulating activity, alpha)	−2.77
6.	CXCL2	Chemokine (C-X-C motif) ligand 2	−3.08
7.	HGF	Hepatocyte growth factor (hepapoietin A; scatter factor)	−7.18
8.	IL2	Interleukin 2	−3.88
9.	ITGA6	Integrin, alpha 6	−4.29
10.	ITGB6	Integrin, beta 6	−5.97
11.	MMP1	Matrix metallopeptidase 1 (interstitial collagenase)	−3.13
12.	PLG	Plasminogen	−4.16
13.	TNF	Tumor necrosis factor	−7.27
14.	WNT5A	Wingless-type MMTV integration site family, member 5A	−2.50
