# Supplementary material for: Evaluating Wharton's Jelly-Derived Mesenchymal Stem Cell's Survival, Migration, and Expression of Wound Repair Markers under Conditions of Ischemia-Like Stress
Source: Stem Cells Int. 2017 Feb 7;2017:5259849. doi: 10.1155/2017/5259849 (PMC5318642; doi:10.1155/2017/5259849)
Supplement: Supplementary file 1 — Effect of individual stress conditions on migration of WJ-MSCs. As ischemic WJ-MSCs exhibited reduced migrational ability compared to control WJ-MSCs, to further investigate the specific stress condition responsible for the decreased migration, WJ-MSCs were cultured under the individual stress conditions and an in vitro scratch assay was performed. [file 5259849.f1.pdf]

Supplemental fig. S1

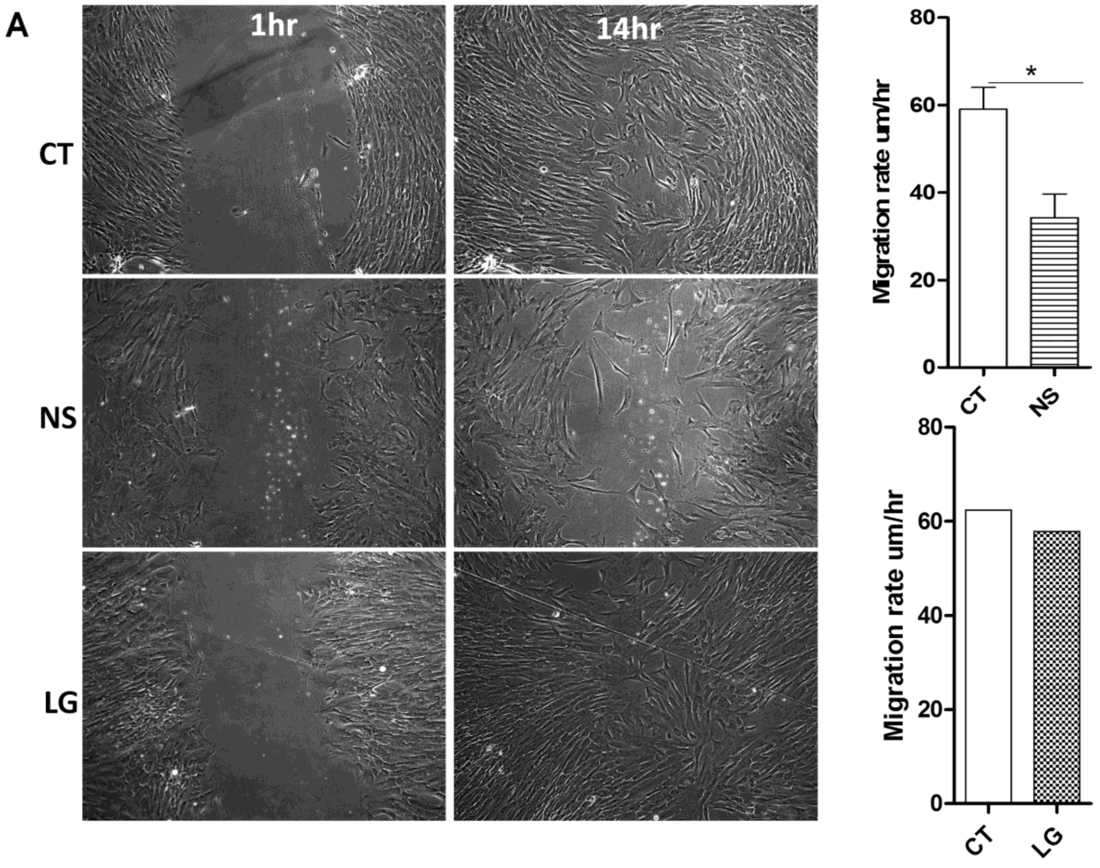

Supplemental fig. S1

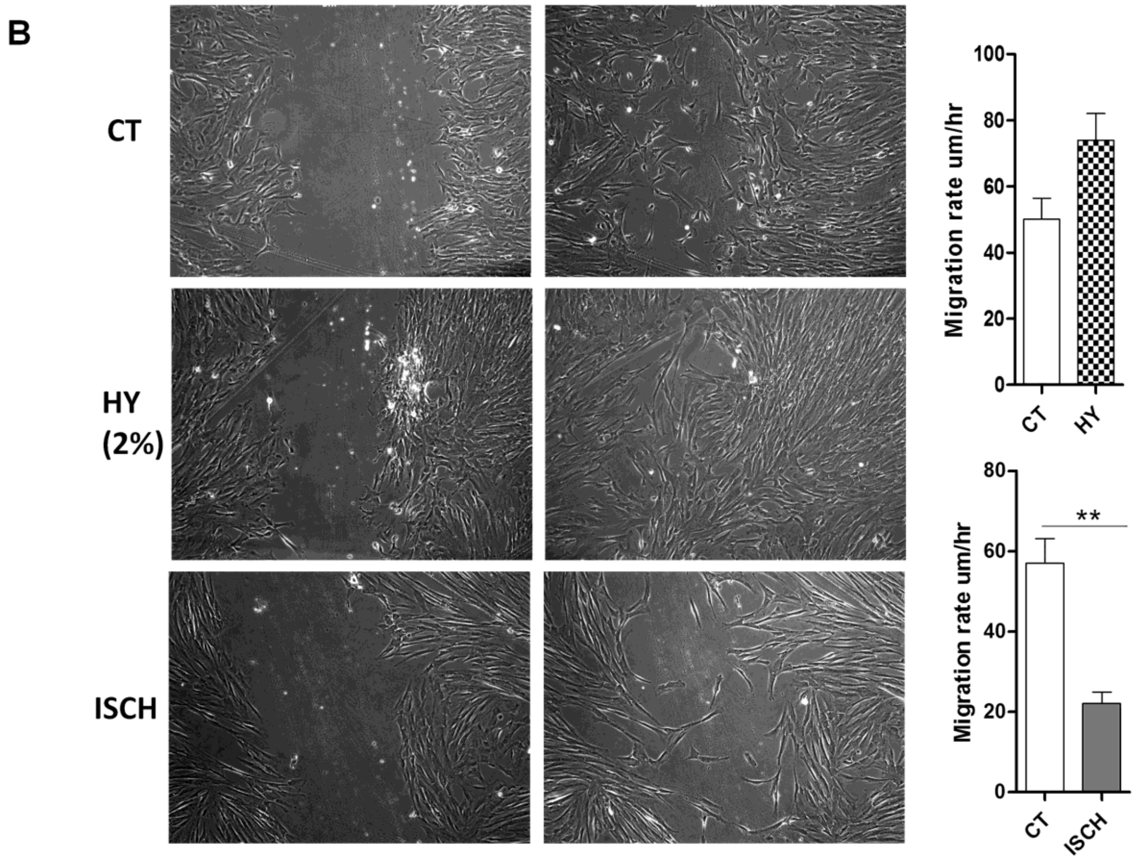

Supplemental Figure S1

Scratch wound healing migration study. Confluent monolayer cultures of WJ-MSCs grown under standard control condition (CT) or individual stress conditions such as no serum containing medium (NS), low glucose medium (LG) (A), and 2% O_2 (HY) and ischemia (which is a combination of low glucose, no serum and 2% O_2) (B) were scratched with a sterile pipette tip at 0hr. Hours since the scratch when images were captured are indicated on images. All images are of 10X magnification. Average migration rate during the first 7-10 hrs in response to the scratch was calculated for two-three independent experiments under each condition. Bars represent mean ±SEM. (Student's t-test, two tailed, * represents $P < 0.05$)
